# Measles during arbovirus outbreak: a diagnostic challenge

**DOI:** 10.1099/jmmcr.0.005156

**Published:** 2018-08-10

**Authors:** Ann-Claire Gourinat, Cécile Cazorla, Anne Pfannstiel, Thomas Tran

**Affiliations:** ^1^​ Centre Hospitalier Territorial de Nouvelle-Calédonie, Microbiology Laboratory, Nouméa, New Caledonia; ^2^​ Centre Hospitalier Territorial de Nouvelle-Calédonie, Infectious Diseases Department, Nouméa, New Caledonia; ^3^​ Gouvernement de la Nouvelle-Calédonie, Direction des Affaires Sanitaires et Sociales, Nouméa, New Caledonia; ^4^​ Regional Measles Reference Laboratory, Victorian Infectious Diseases Reference Laboratory, Melbourne, Australia

**Keywords:** measles, Dengue, viral exanthem, paracetamol, metoclopramide

## Abstract

**Introduction.:**

Dengue fever is a major public health problem in New Caledonia, like in many Pacific Islands Countries and territories. In 2017 New Caledonia faced multiple circulations of arboviruses with a major outbreak of dengue and a co-circulating Zika virus. New Caledonia is considered as a non-endemic territory for measles since the mid 1990’s.

**Case presentation.:**

A 41-year-old male presented fever, headache, sinusitis and exanthematous maculopapular rash. A clinical diagnosis of arbovirus was first suspected due to the local epidemic context. A few days later the patient was admitted to the main hospital. The real time RT-PCR for dengue and Zika virus were negative on the first blood sample. A drug reaction with eosinophilia and systemic symptoms (DRESS) syndrome and other infectious diseases including measles were then suspected. ELISA tests for measles were positive for IgM and equivocal for IgG. A throat swab was immediately shipped to a reference laboratory for measles nucleic acid testing. After a week, the patient recovered and the presence of measles RNA was confirmed. No secondary cases were reported among contacts of the patient and the source of his infection could not be ascertained.

**Conclusion.:**

Diagnosis of measles during an arbovirus outbreak in a country where measles disease is rare can be a pitfall for healthcare professionals. The introduction of measles via returned travellers or tourists from areas where measles remains endemic is a real threat to countries with high vaccine coverage.

## Introduction

The emergence of arboviruses, such as dengue virus (DENV), chikungunya virus (CHIKV) and Zika virus (ZIKV) in the Pacific Islands in the past 10 years, has resulted in an increased number of arbovirus epidemics in New Caledonia [1]. Dengue outbreaks have been a major public health problem since World War II in this French overseas territory and have involved all four DENV serotypes (DENV-1 to DENV-4) [2, 3].

In 2017 New Caledonia experienced multiple outbreaks from co-circulation of arboviruses. These outbreaks lead to 2500 laboratory-confirmed cases of dengue where DENV-1 (78 %) was the major cause of outbreaks followed by DENV-2 (17 %) and DENV-3 (4 %) [4]. Zika virus transmission was also detected by the surveillance network with 38 cases laboratory confirmed by real time RT-PCR [4].

The last large outbreak of measles in New Caledonia occurred in 1987 with more than 1200 cases and 19 cases of subacute sclerosing panencephalitis [5, 6]. Since then, an effective vaccination plan has been established by the health authorities. The introduction of MMR first dose vaccine in 1987 and MMR second dose vaccine in 1992 has resulted in vaccine coverage of 96.4 and 85.6 % for first and second dose respectively [7]. The high vaccine coverage established in New Caledonia has eliminated large measles outbreaks with only the occasional sporadic cases seen. Since the year 2000, only two cases of measles have been detected, of which one was imported from Vanuatu.

Here, we report a case of measles in a patient initially diagnosed clinically as dengue and then as drug reaction and we highlight the difficulty of clinical diagnosis of measles during a major outbreak of dengue.

## Case report

A 41-year-old male living in New Caledonia without significant past medical history and no recent travel, presented to his general practitioner with fever (38.8 °C), headache, sinusitis and exanthematous maculopapular rash over neck, thorax and upper limbs. The symptoms had started 2 days prior and a diagnosis of an arbovirus infection was first considered by the physician due to the current DENV outbreak and a confirmed diagnosis of the patient’s wife of dengue infection by RT-PCR the week before. A few days later a decline of health was observed and the patient was sent to the emergency department, 6 days post the onset of symptoms. On admission, the patient presented with a temperature of 40.5 °C, a spO_2_92 %, a blood pressure of 110/50 mm Hg, dehydration, a weight loss of 6 kg, an erythroderma on trunk and face and an axillary and a cervical lymphadenopathy (<1 cm). A complete blood count showed a lymphopenia (lymphocytes count 0.68×10^9^ l^−1^; reference range 1−5×10^9^ l^−1^) with associated stimulated lymphocytes, and hepatic cytolysis (aspartate aminotransferase 288 IU l^−1^; reference value <34 IU l^−1^, and alanine aminotransferase 485 IU l^−1^; reference value <55 IU l^−1^). The patient was placed on intravenous paracetamol at a dose of 1 g and 10 mg of metoclopramide. The blood sample tested 3 days prior for dengue, chikungunya and Zika by real time RT-PCR was negative for the three arboviruses. With regard to the exanthema, the high fever, a recent administration of ibuprofen, amoxicillin and clavulanic acid and the cytolic hepatitis, a drug reaction with eosinophilia and systemic symptoms (DRESS) syndrome was first considered and the patient was admitted to the internal medicine and infectious disease ward.

## Investigations

New laboratory investigations show a thrombocytopenia (85×10^9^ l^−1^; reference range 150–400×10^9^ l^−1^). Serological assays for hepatitis A, B, C virus, parvovirus B19, human immunodeficiency virus, Epstein–Barr virus and cytomegalovirus were all negative. Koplik spots were not present on the buccal mucosa during the clinical examination but a measles serology was requested due to no history of past measles immunization and the onset of rash.

## Diagnosis

**Fig. 1.**
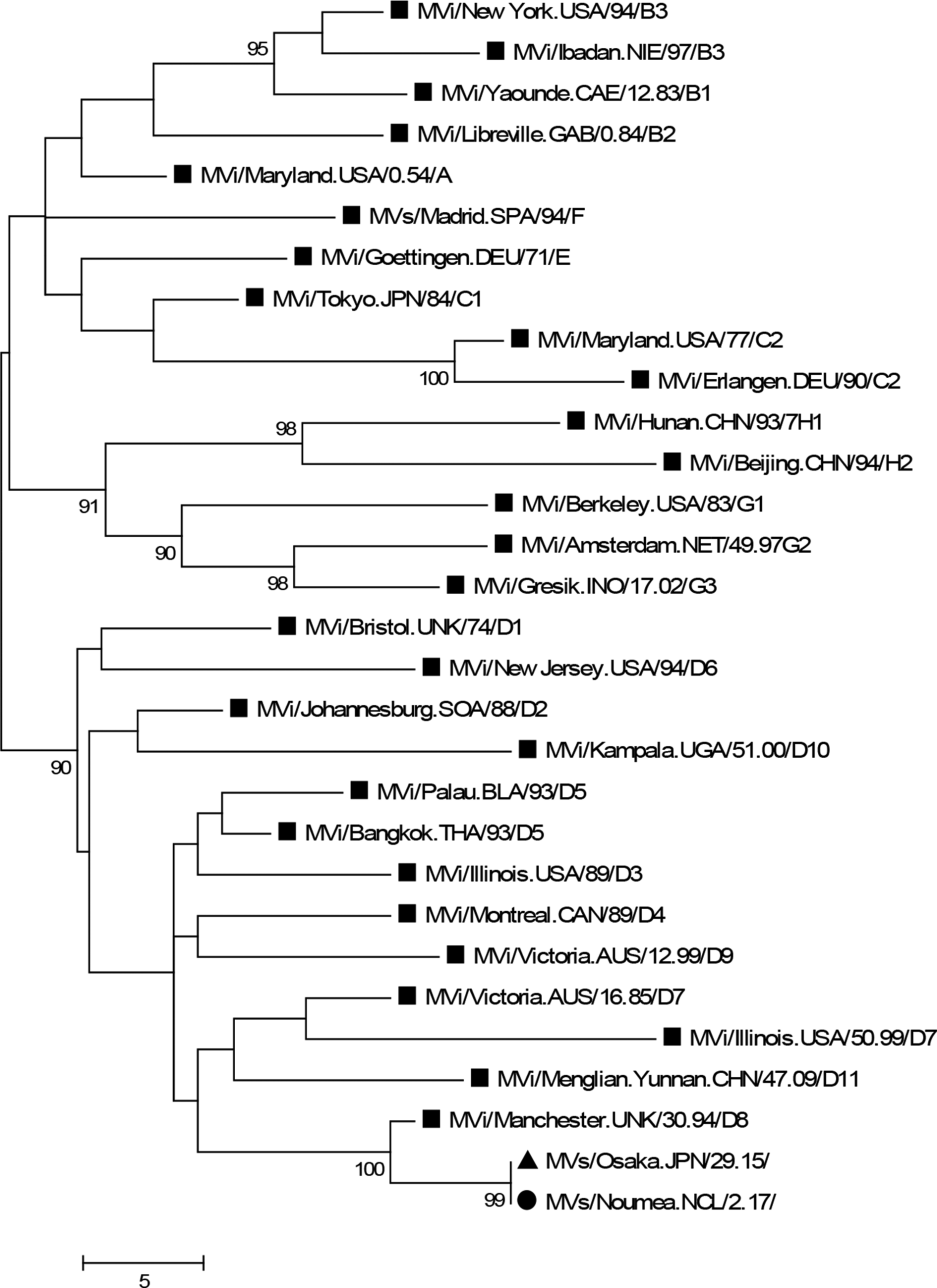
Phylogenetic tree of the New Caledonia measles case (black circle). Black squares: WHO measles reference strains. Black triangle: a prevelant circulating measles genotype D8 strain. The distance-based phylogenetic tree was reconstructed by neighbour-joining methods using the Kimura two-parameter model of evolution with optimised parameters based on 450 nucleotide nucleoprotein sequences. Bootstrap values greater than 90 % are indicated.

ELISA test assays for measles antibodies were positive for IgM and equivocal for IgG (Euroimmun). A throat swab was immediately collected and sent to the Regional Measles Reference Laboratory at the Victorian Infectious Diseases Reference Laboratory in Melbourne, Australia for nucleic acid testing. The real time RT-PCR confirmed the presence of measles RNA in the respiratory specimen. Measles genotyping in our patient revealed the measles D8 genotype (GenBank Accession number MG925077), which fell into the Osaka lineage (Fig. 1).

## Outcome and follow up

After a week, the patient recovered but a bilateral keratitis was diagnosed, followed by treatment with eye drops and normal vision was restored after a few weeks.

No secondary cases were reported among contacts of the patient and the origin of his infection had not been identified by health authorities.

## Discussion

Determining the viral cause of a rash presents significant diagnostic challenges. Important clues to the diagnosis of viral exanthems include their distribution and morphology, geographic location and potential exposure to vector-borne or blood-borne viruses.

Although general practitioners encounter morbilliform eruptions regularly, the cause can often be difficult to ascertain, especially during arbovirus outbreaks. On examination, Koplik spots have to be identified accurately on the buccal mucosa as they are present in more than 70 % of the patients with measles [8].

Serology plays an important part in the diagnosis and the screening of patients for common infections, such as measles, should be considered during an arbovirus outbreak as this infection still remains endemic in several parts of the world even if it is not in New Caledonia.

Molecular analysis of measles virus strains is also an important tool to track the transmission pathways of measles virus genotypes and can be useful in order to determine transmission root. In this case report, the origin of the viral infection was not elucidated by health authorities but the results of the phylogenetic analysis indicated that the PCR sequences belonged to an Osaka named strain. (MVs/Osaka.JPN/29.15). The circulation of this strain has recently been detected in many countries including Italy, Brazil and Japan [9–11], and we can conclude that the virus was imported by a traveller.

Measles remains one of the leading causes of death among young children worldwide despite the availability of a safe and effective vaccine. According to WHO, approximately 89  780 people died from measles in 2016 mostly children under the age of 5 years [12].

Recently it has been observed that the source of global transmission can be from both countries where measles is endemic and those that have eliminated the disease (apart from occasional outbreaks) [13].

In conclusion, the diagnosis of measles during an arbovirus outbreak, in a country where the disease occurs extremely rarely, is challenging for healthcare professionals given that most clinicians may never have encountered patients with measles.

## References

[1] Cao-Lormeau VM, Musso D (2014). Emerging arboviruses in the Pacific. Lancet.

[2] Descloux E, Mangeas M, Menkes CE, Lengaigne M, Leroy A (2012). Climate-based models for understanding and forecasting dengue epidemics. PLoS Negl Trop Dis.

[3] Perry WJ (1950). The mosquitoes and mosquito-borne diseases on New Caledonia, an historic account: 1885–1946. Am J Trop Med.

[4] Direction des Affaires Sanitaires et Sociales de Nouvelle-Calédonie www.dass.gouv.nc/portal/page/portal/dass/observatoire_sante/veille_sanitaire/Dengue.

[5] New Caledonia Country Profile Measles Elimination www.wpro.who.int/immunization/documents/measles_country_profile_may2016_nec.pdf.

[6] Gagnaire JP (2004). Panencéphalite sclérosante subaiguë: à propos de 19 cas secondaires à une épidémie de rougeole en Nouvelle-Calédonie.

[7] Pfannstiel A (2012). Couverture vaccinale des enfants âgés de 4 ans en Nouvelle-Calédonie en 2011.

[8] Lefebvre N, Camuset G, Bui E, Christmann D, Hansmann Y (2010). Koplik spots: a clinical sign with epidemiological implications for measles control. Dermatology.

[9] Amendola A, Bianchi S, Frati ER, Ciceri G, Faccini M (2017). Ongoing large measles outbreak with nosocomial transmission in Milan, northern Italy, March-August 2017. Euro Surveill.

[10] Yagi Y, Higashino H, Yoshida H, Hirokawa H, Okumachi A (2015). The 2014 measles outbreak in Osaka an epidemiological study for the elimination of measles. Nihon Koshu Eisei Zasshi.

[11] Lemos DR, Franco AR, de Sá Roriz ML, Carneiro AK, de Oliveira Garcia MH (2017). Measles epidemic in Brazil in the post-elimination period: coordinated response and containment strategies. Vaccine.

[12] WHO Measles. www.who.int/mediacentre/factsheets/fs286/en/.

[13] Furuse Y, Oshitani H (2017). Global transmission dynamics of measles in the measles elimination era. Viruses.

